# Supplementary material for: 3D Tumor Models in Urology
Source: Int J Mol Sci. 2023 Mar 25;24(7):6232. doi: 10.3390/ijms24076232 (PMC10094462; doi:10.3390/ijms24076232)
Supplement: Supplementary file 1 [file ijms-24-06232-s001.zip › ijms-2300973-supplementary.pdf]

Table S1. Antibodies used for immunofluorescence.

Target	Host	Type	Source	Catalog-No.
**Primary antibodies**				
Actin-beta (ACTB)	Ms	IgG1, monoclonal	[1]	A2228
Ki-67	Rb	IgG, polyclonal	[2]	ab15580
Cytokeratin 5&6 (CK5+6)	Ms	IgG1, monoclonal	[2]	ab17133
Cytokeratin Pan-Alexa Fluor 488 (CKPAN)	Ms	IgG1, monoclonal	[3]	ABIN909639
Ki-67	Ms	IgG1, monoclonal	[4]	MSK018-05
Zonula occludens protein (ZO-1)	Ms	IgG1, monoclonal	[5]	610966
Vinculin (VCL)	Ms	IgG, monoclonal	[1]	V9131
Von Willebrand factor (VWF)	Rb	IgG	[6]	A 0082
**Secondary antibodies**				
anti-Mouse IgG (H+L) Alexa Fluor 488	Gt	IgG	[7]	A32727
anti-Rabbit IgG (H+L) Alexa Fluor 555	Gt	IgG	[7]	A32732
F(ab')2 Fragment of Goat Anti Mouse IgG (H+L)	Gt	IgG	[8]	A11017
594 F(ab')2 Fragment of Goat Anti Rabbit IgG (H+L)	Gt	IgG	[8]	A11072

Rb = rabbit, Gt = goat, Ms = mouse
[1] Sigma-Aldrich, St.-Louis, MI, USA
[2] Abcam, Cambridge, MA, USA
[3] antibodies-online, Aachen, Germany
[4] Zytomed-Systems, Oxfordshire, UK
[5] BD biosciences, Heidelberg, Germany
[6] Agilent (DAKO), Santa Clara, CA, USA
[7] ThermoFisher Scientific (Invitrogen), Rockford, IL, USA
[8] Life Technologies/Invitrogen Karlsbad, CA, USA
